# Prevalence of adhd in the adult population in the Czech Republic and frequency of medication

**DOI:** 10.1192/j.eurpsy.2021.1040

**Published:** 2021-08-13

**Authors:** M. Vňuková, R. Ptáček

**Affiliations:** Psychiatry, First Faculty of Medicine Charles University in Prague and General Teaching Hospital, Prague, Czech Republic

**Keywords:** adult ADHD prevalence, ASRS, medication

## Abstract

**Introduction:**

Attention Deficit Hyperactivity Disorder (ADHD) is a common neurodevelopmental disorder often diagnosed between the ages of 7 and 10. The estimated prevalence of ADHD in adults is usually 2-5%, which means that in up to half of people this diagnosis persists into adulthood.

**Objectives:**

The aim of this sub-study was to determine whether there are significant differences in ADHD symptomatology between individuals who have taken or are taking prescription drugs and those who have never taken them.

**Methods:**

Data collection was performed by STEM / MARK in January 2019 through the European National Panel. Respondents completed a demographic questionnaire focusing on the history of ADHD and a standardized ADHD Self-Report Scale (ASRS) questionnaire for the symptomatology of ADHD in adulthood.

**Results:**

Of the 1,518 respondents, 3% reported being diagnosed with ADHD / hyperkinetic disorder during their lifetime. According to the ASRS assessment, 119 respondents were classified as suspected ADHD. Overall, men scored higher symptoms of ADHD. The results also show that the group that states that taking medication has a statistically significantly higher average score in ASRS than unmedicated individuals. 6 individuals are taking medication to this day.

**Conclusions:**

The data collected confirm our main hypothesis that ADHD symptomatology has an impact on the daily functioning of individuals in adulthood. Unlike peers with reduced or no ADHD symptoms, these people are far more likely to have time management issues, need to plan their day carefully, and yet often experience problems such as late arrivals due to a lack of anticipation.

**Conflict of interest:**

Financial support was provided by grants : GA ČR – 18 -112 47 S, Progres Q06 1LF

